# Theabrownin from Pu-erh Tea Restores Intestinal Immunity via Gut Microbiota–Metabolite Network in Immunosuppressed Mice

**DOI:** 10.3390/foods15193381

**Published:** 2026-09-23

**Authors:** Lei Zhao, Anding Zhu, Jinping Chen, Minglong Shao, Jinhuan Zan, Jiashun Gong, Song Li

**Affiliations:** 1 College of Science, Yunnan Agricultural University, Kunming 650201, China; zhaolei928@ynau.edu.cn (L.Z.); z13330504060@163.com (A.Z.); m19187795662@163.com (J.C.); m13638820203@163.com (M.S.); z3259873617@163.com (J.Z.); 2 Agro-Products Processing Research Institute, Yunnan Academy of Agricultural Sciences, Kunming 650223, China

**Keywords:** theabrownin, immunosuppression, gut immunity, microbiota–metabolite network, caffeine metabolism, histidine metabolism

## Abstract

Theabrownin (TB), a polymeric pigment derived from ripened Pu-erh tea, demonstrates beneficial effects on lipid reduction, weight management, and mitigation of metabolic syndrome. However, the role of TB in restoring immune function in vivo has not been studied. This study evaluated the therapeutic effects and underlying mechanisms of TB in cyclophosphamide (CTX)-induced immunosuppressed mice. TB was administered at doses of 1.215, 0.608, and 0.304 mg/kg body weight, with levamisole hydrochloride (40 mg/kg) serving as a positive drug control. Each experimental group comprised eight mice. Evaluations primarily consisted of histopathological examination, enzyme-linked immunosorbent assay, Western blotting, flow cytometry, 16S rRNA sequencing, and ultra-performance liquid chromatography–tandem mass spectrometry (UPLC-MS/MS)-based untargeted metabolomics. The results demonstrate that high- and medium-dose TB treatment significantly alleviated immunosuppression, as evidenced by restored body and organ weights, colon length, splenic and colonic histoarchitecture, blood cell counts, and immunoglobulin levels. These improvements correlated with upregulated colonic cytokines, claudin-1, occludin, toll-like receptor 2/4, and reduced regulatory T cell populations. High- and medium-dose TB treatment also enriched beneficial gut microbiota, including *Lactobacillus* and *Candidatus saccharimonas*, accompanied by strong microbiota–metabolite correlations (M^2^ = 0.725, *p* = 0.003). Metabolic KEGG pathway enrichment analysis revealed that moderate-dose TB significantly activated caffeine metabolism and histidine metabolism, accompanied by the enrichment of multiple immune signaling pathways driven by diacylglycerol series lipid metabolites, whereas high-dose TB induced a greater number of immune signaling pathways. Overall, TB may reverse CTX-induced immunosuppression by integrating gut microbiota–metabolite reprogramming, highlighting TB’s potential as an immunomodulatory functional component of medicine-food homologous products.

## 1. Introduction

The gut is a selective barrier that facilitates nutrient absorption while blocking pathogenic microorganisms from entering the body. As a site densely populated with immune cells, it represents a crucial interface linking the gut microbiota to systemic immune responses [1]. Within the intestinal environment, microbes contribute to the development and regulation of the gut immune system, fostering a state of tolerance that permits peaceful coexistence between the microbiota and host cells [2]. However, disruption of the gut microbiota balance can lead to loss of immune tolerance and the development of autoimmune conditions [3,4,5,6]. Therefore, the intricate crosstalk among the microbiota, intestinal barrier, and intestinal immune system is essential for maintaining proper immune function.

Diet can affect changes in gut microorganisms and their metabolites, regulating the intestinal immune system. As early as more than a century ago, in 1906, The Lancet reported that the relative immunity of Chinese people to typhoid fever was stronger than that of Europeans, which was attributed to the widespread tea-drinking custom in China [7]. Tea is one of the most widely consumed beverages globally and is associated with a reduced risk of certain chronic human diseases, including cardiovascular diseases and various cancers [8]. In animal models, bioactive components extracted from tea exhibit anti-inflammatory and antioxidant properties, improve hepatic metabolic function, modulate gut microbiota, reshape intestinal barrier function, and inhibit intestinal inflammation [9,10,11,12,13]. It is important to note that most of these studies have been conducted in rodents. Among the bioactive components of tea, tea polysaccharides [14], theanine [15,16], and tea polyphenols [17,18] have all been demonstrated to exert distinct roles in immune regulation. Unlike whole tea or tea beverages, these purified components are typically administered at specific doses, allowing for a more precise assessment of their specific contributions to immunoenhancement.

Theabrownin (TB) in ripened Pu-erh tea is a polymeric pigment formed through the extensive oxidation of theaflavins, thearubigins, tea polysaccharides, proteins, caffeine, lipids, and other constituents, and it constitutes up to approximately 12% of the dry weight of Pu-erh tea [19,20]. Due to its broad molecular weight distribution [21,22], TB is poorly absorbed directly by the host, making its interaction with the gut microbiota the primary pathway for its biological utilization. Previous studies have demonstrated that TB significantly enriches beneficial gut bacteria and alleviates dextran sulfate sodium-induced colitis [23,24]. However, whether TB can enhance intestinal immunity via modulation of the gut microbiota remains unexplored. Therefore, this study employs a cyclophosphamide (CTX)-induced immunosuppressed mouse model to systematically investigate the relationship between TB, gut microbiota, and intestinal immunity, with a focus on how TB-mediated microbial regulation influences the gut immune system. The findings will advance our understanding of the interplay between diet, gut microbiota, and host health, providing a scientific basis for developing Pu-erh tea-based functional foods or beverages aimed at immune enhancement.

## 2. Materials and Methods

### 2.1. Materials and Reagents

Ripened Pu-erh tea (No.7262, 2018, Taetea Group, Kunming, China), CTX (0.5 g, Hengrui Pharmaceuticals Co., Ltd., Lianyungang, China), levamisole hydrochloride (LH) (Guilin Pharmaceutical Co., Ltd., Guilin, China), and lgG, lgM and lgA enzyme-linked immunosorbent assay (ELISA) kits (Shanghai Enzyme-linked Biotechnology Co., Ltd., Shanghai, China) were obtained. Primary antibodies, toll-like receptor 2 (TLR2), TLR4, mediated myeloid differentiation factor 88 (MyD88), claudin-1, occludin, tumor necrosis factor α (TNF-α), interleukin 6 (IL-6), IL-1β, inducible nitric oxide synthase (iNOS), and β-actin, as well as HRP-conjugated goat anti-rabbit/mouse recombinant secondary antibodies and an enhanced chemiluminescence (ECL) detection kit, were purchased from Proteintech (Wuhan, China). Flow cytometry antibodies, including CD25-PE (BD Biosciences, San Jose, CA, USA) and CD4-FITC/IL-17A-PE/FOXP3-APC (Thermo Fisher Scientific, Waltham, MA, USA), were used. RIPA buffer(high), 5 × Tris-Glycine Running Buffer, 20 × TBST, BCA protein assay kit, antibody dilution buffer, and stripping buffer were purchased from Solarbio (Beijing, China). Other reagents were of analytical grade.

### 2.2. Preparation of TB

The TB used in this study was the remaining portion of the same preparation described in our previous work [23]. Briefly, TB was extracted as reported therein, and its structural characteristics were confirmed to be consistent with those described in reference [23]. The extraction yield and purity of the TB preparation used here were identical to those reported in reference [23] (purity: 96.58%; yield: 10.56%).

### 2.3. Animal Experiment Design

Specific pathogen-free (SPF)-grade ICR male mice (5–6 weeks, weighing 30 ± 3 g) were used in this study and were supplied by Henan Skobes Biotechnology Co., Ltd. (Anyang, China, certificate: SCXK (Yu)-2020-0005). This study was approved by the life science ethics committee of Yunnan Agricultural University (No: 202305006). All mice were housed in the animal facility laboratory of the College of Food Science, Yunnan Agricultural University (Kunming, China), under controlled conditions (temperature: 22–25 °C; 12 h light/dark cycle) with ad libitum access to standard rodent chow and ultrapure water. All mice were randomly divided into 6 groups (*n* = 8) after one week of acclimatization: CON group (normal mice), CTX group (immunosuppressive model), LH group (LH 40 mg/kg + CTX), TBH group (TB 1.215 g/kg + CTX), TBM group (TB 0.608 g/kg + CTX), and TBL group (TB 0.304 g/kg + CTX).

From Day 1 to Day 21, TBH, TBM, and TBL groups received intragastric administration of the corresponding TB preparations (200 μL per mouse). The CON, LH, and CTX groups received an equal volume of PBS as a vehicle control. During Days 22–24, gavage was discontinued in all groups, and mice in the CTX, LH, TBH, TBM, and TBL groups received intraperitoneal injections of CTX (80 mg/kg) once daily for three consecutive days to establish the immunosuppressed mouse model. From Day 25 to Day 31, the LH, TBH, TBM, and TBL groups received the respective treatments. At the end of the experiment, blood samples were collected, and mice were sacrificed by cervical dislocation. The animal experiment was conducted according to the protocol shown in Figure 1.

### 2.4. Body Weight and Organ Index Measurement

Body weight of mice in each group was measured daily. After euthanasia, the spleen and thymus were excised, blotted dry with filter paper to remove residual blood, and weighed. The organ index was calculated using the following formula:

$$\mathrm{Organ}\mathrm{index}(mg/g)=\frac{\mathrm{organ}\mathrm{weight}}{\mathrm{body}\mathrm{weight}}\times 1000$$

### 2.5. Histological Analysis

Colon and spleen specimens were fixed in 4% formaldehyde for 24 h before sectioning and hematoxylin and eosin(H&E) staining. The morphology of the staining was observed by an inverted microscope (Leica, Tokyo, Japan).

### 2.6. Peripheral Blood Cells Analysis

Mouse blood samples were collected in 2 mL EDTA-K3 anticoagulant tubes, and a complete blood count (CBC) was analyzed using an automated hematology analyzer (Mindray BC-20, Shenzhen, China).

### 2.7. Measurement of Immunoglobulin in Serum

The serum concentrations of lgG, lgM and lgA were measured using commercial ELISA kits according to the manufacturer’s instructions.

### 2.8. Flow Cytometry

The lamina propria lymphocytes were isolated from the colon following the established protocol by Fan et al. [25]. The cell suspension was adjusted to a concentration of 1 × 10^7^ cells/mL and subsequently stained with fluorescently labeled antibodies for flow cytometric analysis of Th17 and Treg cell populations: Th17 cells were identified as CD4 (FITC) and IL-17A (PE) double-positive cells, while Treg cells were defined as CD4 (FITC), CD25 (PE), and Foxp3 (APC) triple-positive populations. Finally, data acquisition was performed using a FACS Calibur flow cytometer (BD Biosciences).

### 2.9. Western Blotting (WB)

Protein extraction from colonic tissues (0.1 g) was performed using RIPA lysis buffer, with concentrations determined by BCA assay. Samples (30 μg/lane) underwent electrophoretic separation on 7.5%~10% SDS-PAGE gels (170 mV), followed by transfer to PVDF membranes. After blocking with 5% skim milk (1–2 h), membranes were incubated with primary antibodies (IL-1β, IL-6, IL-10, TNF-α, iNOS, occludin and claudin-1, TLR2, TLR4, MyD88) (4 °C, overnight) and corresponding HRP-conjugated secondary antibodies (room temperature, 1 h). All steps were interspersed with two TBST washes (10–15 min each). Finally, the target protein bands were visualized using ECL detection on Chemidoc touch imaging (Bio-Rad, Hercules, CA, USA). After washing PVDF in stripping buffer for 30 min, reseal it and incubate with other primary antibodies or β-actin. The subsequent steps were the same as before.

### 2.10. 16S rRNA Sequencing and Gut Microbiota Analysis

Microbiota genomic DNA was extracted from cecal contents using a FastPure stool DNA isolation kit (MJYH, Shanghai, China). DNA concentration and purity were assessed by 1% agarose gel electrophoresis. The V3–V4 hypervariable region of 16S rRNA genes was amplified using primers 338F/806R. Qualified libraries were sequenced on an Illumina NovaSeq 2000 platform. The gut microbiota analysis was conducted by Majorbio Bio-Pharm Technology Co., Ltd. (Shanghai, China), with subsequent data processing through their Majorbio Cloud platform (cloud.majorbio.com). The raw sequencing reads were deposited into the NCBI Sequence Read Archive (SRA) database (PRJNA1455889).

### 2.11. Non-Targeted Metabolomics Analysis

The cecal contents were processed according to established protocols [26]. Metabolite profiling was performed using an ultra-high-performance liquid chromatography (UHPLC)-Q Exactive HF-X system (Thermo Fisher). The LC-MS/MS analysis was conducted by Majorbio Bio-Pharm Technology Co., Ltd. (Shanghai, China), with subsequent data processing through the Majorbio Cloud platform (cloud.majorbio.com). Both chromatographic and mass spectrometric conditions followed standard analytical parameters.

### 2.12. Data Analysis and Graphic Drawing

Statistical comparisons were performed using one-way ANOVA in GraphPad Prism (v8.0.2), with data presented as mean ± SD (statistical significance set at *p* < 0.05). All bar graphs were generated using GraphPad Prism v11.1.0. Flow cytometry data were processed with FlowJo (v10), while WB images were quantified using ImageJ (v1.53k). The figures were created using Microsoft PowerPoint software (2021).

## 3. Results

### 3.1. TB Ameliorates CTX-Induced Systemic Immunosuppression

The intake of TB can effectively protect mice from CTX-induced immunosuppression, as evidenced by significant improvements in body weight, organ indices, peripheral blood cell counts, and intestinal barrier integrity.

#### 3.1.1. Effects of TB on Body Weight and Organ Index

Body weight changes were monitored starting after CTX injection. As shown in Figure 2A, three days after CTX injection, body weight began to decline in all groups except the CON group. By the end of the experiment (Day 31st) (Figure 2B), the CTX group showed a significant reduction in relative body weight (85.35 ± 2.51%). But all TB treatment groups effectively maintained body weight, with performance comparable to the LH-positive control.

Following CTX administration, a marked reduction in thymus size was observed. As illustrated in Figure 2C, the thymic index decreased significantly in the CTX group, whereas all TB-treated groups exhibited a notable recovery in thymic mass. In contrast to the spleen atrophy reported in some studies [27], we observed CTX-induced splenomegaly in this model, with the spleen index showing an increasing trend (Figure 2D,E). The CTX group reached a splenic index of 8.99 ± 1.43 mg/g, while TB treatment at all doses significantly attenuated this enlargement.

#### 3.1.2. Effects of TB on Peripheral Blood Cells and Immunoglobulins

CTX induces a significant reduction in white blood cell (WBC) count in the peripheral blood of mice. As illustrated in Figure 2F, the CTX group showed a 39.84% decrease in WBCs compared to the CON group. Specifically, granulocytes decreased by 51.72% (Figure 2G), lymphocytes by 36.97% (Figure 2H), and monocytes by 42.86% (Figure 2I). Meanwhile, medium and high doses of TB significantly increased platelet count (Figure 2J), whereas red blood cell (RBC) counts and hemoglobin levels remained unchanged (Figure 2K,L). These results demonstrate that TB administration protects against CTX-induced leukocyte and platelet loss, suggesting an immunoprotective effect.

As shown in Figure 2M–O, serum immunoglobulin levels in the CTX group were significantly reduced relative to the CON group: IgG was 45.85% lower, IgA was 28.52% lower, and IgM was 35.21% lower. In contrast, all TB-treated groups exhibited elevated levels of IgG, IgA, and IgM, with no statistically significant differences observed compared to either the CON or LH groups.

#### 3.1.3. Effects of TB on Spleen and Colon

The histological analysis of spleen sections stained with H&E (Figure 3A–F) revealed that mice in the CON group exhibited an intact splenic capsule, well-demarcated boundaries between the red pulp (RP) and white pulp (WP), and an absence of reactive hyperplasia or atrophy in the WP lymphoid follicles (Figure 3A). But CTX treatment induced significant pathological alterations in the spleen, including the presence of extramedullary hematopoiesis and marked atrophy of the WP (Figure 3B). Notably, administration of high- and medium-dose TB formulations effectively ameliorated WP atrophy, restored clear RP-WP demarcation, and substantially improved the splenic immune microenvironment (Figure 3D,E).

CTX administration shortened the colon length in mice, a pathological change that was ameliorated by TB treatment (Figure 3G,H). Histological analysis of H&E-stained colon sections (Figure 3I–N) revealed that CTX led to a decrease in goblet cells in the colon epithelial cells (Figure 3J), which are crucial for mucin secretion and intestinal homeostasis. Importantly, TB treatment at all doses increased the number of goblet cells in the colon epithelium.

Furthermore, CTX administration significantly inhibited the expression of tight junction proteins (TJPs), including claudin-1 and occludin, in the intestine (Figure 3O–Q). Effective reversal of this inhibition was observed following high/medium-dose TB treatment, suggesting that TB may contribute to the maintenance of intestinal tight junction structural integrity.

### 3.2. TB Modulates Colonic Immune Microenvironment

To further investigate the improvement of TB on systemic immunosuppression in CTX-induced mice, the levels of pro-inflammatory mediators, expression of toll-like receptors, and differentiation status of CD4^+^ T cells in the lamina propria of the colon were further evaluated with a focus on the primary metabolic sites of TB. These analyses aimed to determine whether TB exerts a regulatory role in intestinal mucosa and gut immunity.

#### 3.2.1. Effects of TB on Pro-Inflammatory Mediators in Colon

Under immunosuppressed conditions, pro-inflammatory mediators are typically downregulated, whereas anti-inflammatory cytokines such as IL-10 are elevated. In the present study, protein extracts from colon tissues were analyzed by WB for the expression levels of IL-6, IL-1β, TNF-α, IL-10, and iNOS (Figure 4A–F). The CTX group exhibited marked reductions in IL-6, IL-1β, TNF-α, and iNOS, accompanied by an increase in IL-10, consistent with a state of immunosuppression. All TB dosage groups effectively counteracted these alterations, with high- and medium-dose treatments demonstrating superior efficacy to the low-dose group.

#### 3.2.2. Effects of TB on Colonic Toll-like Receptors (TLRs)

TLR expression in the colon is intimately associated with intestinal immunity [28]. The results indicated that CTX reduced the expression of TLR2 and TLR4 in colonic epithelial cells, along with a decrease in the intracellular adaptor protein MyD88 (Figure 4G). This suppression implies impaired MyD88-dependent signaling cascades, such as MAPK and NF-κB pathways, potentially contributing to the observed cytokine reductions [29,30]. Both TBH and TBM treatments significantly rescued the expression of TLR2, TLR4, and MyD88 compared to the CTX group (Figure 4G–J). TB-mediated regulation remained moderate, avoiding excessive inflammatory activation and suggesting a balanced immunomodulatory role.

#### 3.2.3. Effects of TB on CD4^+^ T Cell Differentiation in the Colonic Lamina Propria

CTX causes abnormal differentiation of CD4^+^ cells [31]. As illustrated in Figure 5B, induction of CTX significantly increased the proportion of Treg cells. All TB dosage groups effectively reduced Treg levels (Figure 5D–G), with the TBH group showing values comparable to those in the CON group (Figure 5A). LH group did not exert an effect on reducing the proportion of Treg cells (Figure 5C). Concurrently, the differentiation of Th17 cells was evaluated (Figure 5H–N). No significant differences were observed between the CTX and TBH/TBM groups. The differentiation ratio of Th17 and Treg cells is illustrated in Figure 5O. Although Th17 proportions did not differ significantly across groups, a shift in the Th17/Treg balance was observed. Specifically, TBH treatments promoted a trend toward Th17 predominance. Restoration of the Th17/Treg equilibrium contributes to the amelioration of autoimmune responses under TBH treatment.

### 3.3. TB-Induced Microbiota–Metabolite Network and Immune Restoration

TB reshapes the gut microbiota composition and reprograms the metabolic landscape in immunosuppressed mice. Integrative network analysis further points to a close relationship between TB-enriched bacterial taxa and immunomodulatory metabolites, suggesting that this microbiota–metabolite network may be involved in TB-mediated intestinal immune protection.

#### 3.3.1. Effects of TB on CTX-Induced Intestinal Microbiota Alterations

TB can positively modulate the gut microbiota in mouse models of various metabolic disorders [32]. In this study, we assessed the α-diversity of the gut microbiota of the cecum in CTX-induced immunosuppressed mice following TB treatment using the Chao1 index (Figure 6A) and Simpson index (Figure 6B). From the Chao 1 index, there were no significant differences among the groups. However, from the Simpson index, the value of the TBM group was significantly higher than those of the CON group, CTX group, and TBL group, suggesting that intervention with a moderate dose of TB can significantly increase the relative abundance distribution uniformity of the microbiota. β-Diversity was employed to evaluate similarities or differences in overall microbial community structure. Principal coordinates analysis (PCoA) (Figure 6C) revealed that the microbial composition in groups receiving high- and medium-dose TB closely resembled that of the LH-positive control group. In contrast, low-dose TB had a comparatively modest impact on the gut microbiota. Venn diagrams illustrating the distribution of operational taxonomic units (OTUs) across experimental groups are presented in Figure 6D.

Based on the phylum-level composition of the microbiota (Figure 6E), the TBH, TBM, and LH groups increased the relative abundance of *Firmicutes* (*F*) and reduced that of *Bacteroidetes* (*B*), leading to a significant rise in the *F*/*B* ratio (Figure 6F). Figure 6G displays the evolutionary branching relationships among microbial communities across groups. Meanwhile, Figure 6H presents the top 10 bacterial genera exhibiting the most pronounced differences in abundance. Specifically, TBH/M significantly enhanced the abundance of *Lactobacillus*, with the TBH group averaging 29.99% and TBM reaching 42.82% respectively (Figure 6H). Differential abundance analysis between the TBH and CTX groups showed that TBH significantly increased the abundance of *Lactobacillus*, *Candidatus_Saccharimonas*, *Romboutsia*, *Clostridium_sensu_stricto_1*, and *Jeotgalicoccus*, with the first two genera exhibiting the most pronounced increases (Figure 6I). A similar comparison between the TBM and CTX groups revealed consistent trends for *Lactobacillus* and *Candidatus_Saccharimonas* (Figure 6J).

#### 3.3.2. Effects of TB on CTX-Induced Intestinal Metabolite Alterations

The metabolomics across experimental groups were evaluated using partial least squares discriminant analysis (PLS-DA). As shown in Figure 7A, there was overlap among the groups. In contrast, samples from the CTX and TBH groups exhibited clustering separation on the score plot, suggesting that TBH interventions significantly altered the intestinal metabolome induced by CTX in mice (*R*^2^Y = 0.994, *Q*^2^ = 0.827, *p* < 0.05) (Figure 7B). Figure 7C presents a Venn diagram illustrating the differential metabolites identified in CTX compared with those in TBH and TBM. A total of 133 metabolites were common across all groups, while TBH and TBM exhibited 199 and 373 unique differential metabolites, respectively. Further analysis showed that among the most significantly altered metabolites in TBM versus CTX, lipids and lipid-like molecules accounted for 31.12%, followed by organoheterocyclic compounds (19.39%), and organic acids and derivatives (16.07%) (Figure 7D). Similarly, in the TBH versus CTX comparison, lipids and lipid-like molecules represented 38.11% of the top differential metabolites, with organoheterocyclic compounds comprising 17.62%, and organic acids and derivatives accounting for 16.39% (Figure 7E). These results indicate that high-dose TB leads to a greater abundance of lipids and lipid-like molecules among microbial metabolites in the intestine. In addition, volcano plots of metabolites from the TBM versus CTX comparison (Figure 7F,G) revealed 191 upregulated and 315 downregulated metabolites. In contrast, the TBH versus CTX comparison (Figure 7H,I) showed 200 upregulated and 132 downregulated metabolites.

Screening with a variable importance in projection (VIP) score ≥ 4 identified four differential metabolites in the TBM versus CTX comparison, including an upregulated 6-hydroxy-2-cyclohexen-one-carboxylate (Figure 7J). In the TBH versus CTX comparison, this screening identified 14 upregulated metabolites (Figure 7K). According to Kyoto Encyclopedia of Genes and Genomes (KEGG) enrichment analysis of differential metabolites, we examined the top 20 significantly enriched signaling pathways in the TBM versus CTX group (*p* < 0.05, Figure 7L). Caffeine metabolism (*p*_adjust = 0.001876) and histidine metabolism (*p*_adjust = 0.003955) were the only two pathways that remained significantly activated after false discovery rate (FDR) correction. Multiple immune-related pathways (e.g., Fc epsilon RI, NF-κB, Th1/Th2, Th17) exhibited an activation trend (*p* < 0.05, but *p*_adjust > 0.05), primarily driven by diacylglycerols (Dg)-series lipid metabolites Dg (22:4/18:2), whose biological significance requires further validation. The TBH group displayed a pattern similar to that of the TBM group relative to CTX, yet only caffeine metabolism survived FDR correction (*p*_adjust = 5.30 × 10^−5^). More immune-related pathways, driven by lipid metabolite Dg lipid and histamine, were observed in the TBH group compared to the TBM group, such as natural killer cell-mediated cytotoxicity, aldosterone synthesis and secretion.

#### 3.3.3. Effects of TB on Gut Microbiota–Metabolite Network

Spearman’s rank correlation test and Procrustes analysis both pointed to a strong agreement between the gut microbiome and metabolome profiles (M^2^ = 0.725, *p* = 0.003) (Figure 8A). This suggests that in CTX-treated mice, tuberculosis-induced remodeling of the gut microbiota largely drove the accumulation of microbiota-derived metabolites.

A Spearman correlation heatmap depicting associations between the gut microbiota and microbial metabolites in the TBM and CTX groups is shown in Figure 8B. The analysis revealed significant positive correlations between TBM-enriched genera—*Lactobacillus*, *Candidatus_Saccharimonas*, and *Acetitomaculum*—and the metabolites 6-hydroxy-2-cyclohexen-one-carboxylate, 6-amino-5-formamido-1,3-dimethyluracil, and N-acetylhistamine. However, high-dose TB induced more complex alterations in the gut microbiota and microbial metabolites (Figure 8C). Correlation analysis revealed significant positive associations between five TBH-enriched genera—*Lactobacillus*, *Candidatus Saccharimonas*, *Romboutsia*, among others—and 24 metabolites, such as histamine, 1,7-dimethyluric acid, theophylline, and 2-amino-4,6-dinitrotoluene.

## 4. Discussion

The present work establishes that TB, a polymeric pigment derived from ripened Pu-erh tea, exerts pleiotropic immunomodulatory actions in CTX-induced immunosuppressed mice. TB (particularly at medium and high doses) effectively reversed weight loss, colon shortening, and abnormal thymus/spleen indices, significantly alleviated peripheral blood leukopenia, and restored serum immunoglobulin levels. Moreover, TB modulated the expression of colonic pro-inflammatory cytokines, tight junction proteins, and TLR2/4, while also modulating the balance between Th17 and Treg cells in the colonic lamina propria. These findings indicate that TB not only enhances immune responses but also helps prevent excessive inflammation.

CTX is a nitrogen mustard alkylating agent that functions both as a cytotoxic antitumor drug and an immunosuppressant. It is most toxic to rapidly dividing cells, making actively activating and proliferating hematopoietic precursors, B cells, and T cells its primary targets [33,34,35]. There are a large number of immune cells in the spleen. The splenomegaly we previously observed is speculated to be related to EMH. Under physiological conditions, hematopoietic activity in adult murine spleens is minimal, with the bone marrow serving as the primary hematopoietic site. However, under pathological stimuli—such as bone marrow suppression or anemia—the spleen can reactivate hematopoietic functions [36]. An early study on the effects of CTX in mice reported a biphasic response in the spleen: initial atrophy followed by mid-phase enlargement [37]. We hypothesize that CTX, via DNA alkylation, disrupts rapidly proliferating hematopoietic stem cells, impairing bone marrow function and triggering compensatory EMH in the spleen, ultimately leading to splenomegaly. TB treatment significantly attenuated this EMH-associated splenic enlargement, suggesting a protective effect on bone marrow hematopoietic reserves.

Beyond its impact on myelopoiesis, CTX selectively ablates B-cell precursors and mature B cells, thereby blocking their terminal differentiation into plasma cells and interfering with immunoglobulin synthesis, which results in markedly reduced production of IgG, IgA, and IgM [38]. IgG, which includes multiple subtypes, plays a major effector role in humoral immunity. IgA is mainly secreted by mucosa-associated lymphoid tissue and plays a key role in intestinal mucosal immunity, while IgM is critical for initiating early immune responses. These three immunoglobulins are therefore important for maintaining normal immune function. In this study, TB intervention restored the production of IgG, IgA, and IgM, thereby enhancing the body’s immune function.

The intestine is a key site of immune regulation, largely due to the abundance of CD4^+^ T cells in the lamina propria. Within the gut microenvironment (cytokines, bacterial metabolites, and local antigens), CD4^+^ T cells differentiate into various functionally distinct subsets that collectively maintain intestinal immune homeostasis [39]. Th17 cells play a critical protective role in mucosal barrier defense. Tregs produce the anti-inflammatory cytokine IL-10, which helps suppress excessive immune activation and inflammation. However, an overabundance of Tregs can lead to immunosuppression and related pathological changes [40,41]. In this study, TB intervention reduced the proportion of Tregs, appropriately increased the Th17/Treg ratio, and restored balance, thereby effectively counteracting the immunosuppressive state.

Given the complex composition of TB, certain components—including flavonoids, phenolic acids, and polysaccharides—are resistant to digestion in the upper gastrointestinal tract. Consequently, the gut microbiota is likely to play a primary role in the biotransformation of TB. Not only does TB provide these microorganisms with a rich nutrient source, but the metabolites they produce can also be absorbed and utilized by the host. This microbiota-centric perspective prompted us to investigate whether the immunoenhancing efficacy of TB is mediated through microbial action.

At the genus level, both the TBH and TBM groups exhibited the most significant increase in relative abundance for *Lactobacillus* and *Candidatus Saccharimonas*. *Lactobacillus*, a key constituent of the murine gut microbiota, contributes to host health through multiple mechanisms. It colonizes the intestinal mucosa, preventing pathogen adhesion and enhancing epithelial barrier integrity through the upregulation of TJPs, including occludin and claudin-1 [42]. It also supports mucosal immunity by activating gut-associated lymphoid tissue, promoting lgA secretion and strengthening defensive responses, modulating host metabolism, and contributing to immune enhancement [43]. The benefits of *Lactobacillus* are consistent with the results of this study.

From the correlation analysis between the microbiota and metabolites, in the TBH versus CTX group, the increased abundance of *Lactobacillus* was accompanied by synchronous increases in histamine, 1,7-dimethyluric acid, 6-hydroxy-2-cyclohexen-one-carboxylate, and 6-amino-5-formamido-1,3-dimethyluracil (*p*_adjust < 0.05). Tryptamine and 5-p-coumaroylquinic acid also showed positive correlations, but *p*_adjust > 0.05. Among these, histamine and tryptamine are known immunologically active molecules of microbial origin that can influence host inflammatory responses and barrier function. 5-p-coumaroylquinic acid exhibits antioxidant and enzyme inhibitory activities. However, the causal relationship between 6-amino-5-formamido-1,3-dimethyluracil, 6-hydroxy-2-cyclohexen-one-carboxylate and *Lactobacillus* remains unclear, and their biological significance requires further validation. These associations suggest that *Lactobacillus* may participate in the regulation of the intestinal immune microenvironment through its metabolic products. Furthermore, *Candidatus Saccharimonas* has been frequently reported to be correlated with Lactobacillus abundance [44,45,46]. Regarding its function, there are two primary perspectives: one classifies it as a pro-inflammatory bacterium [47,48], while another attributes anti-inflammatory properties to it [49]. Additionally, some studies indicate that this genus contributes to the production and metabolism of short-chain fatty acids, thereby influencing host inflammatory responses [50]. In this study, taking the TBH versus CTX group as an example, an increase in *Candidatus Saccharimonas* abundance was associated with ten elevated metabolites (*p*_adjust < 0.05), including histamine, 1,7-dimethyluric acid, and 6-methylmercaptopurine, which are similar to the metabolites activated by *Lactobacillus*, while 6-methylmercaptopurine is related to drug metabolism. Based on the above analysis, it is hypothesized that the function of *Candidatus Saccharimonas* aligns with that of *Lactobacillus*, enabling the regulation of intestinal immune homeostasis.

Among these microbiota-associated metabolites, Dg-series lipids also warrant attention. DG (22:4/18:2) was present in both the TBM and TBH metabolite pools. Although its VIP value falls between 2 and 3, this Dg family is known to function as signaling intermediates in immune activation. For instance, diacylglycerol (DAG) [51], DG (40:6), and DG (38:4) [52]. These findings suggest that Dg lipids are not merely metabolic byproducts but functional intermediates capable of actively modulating immune signal transduction.

A comprehensive analysis of the microbiota–metabolite network indicates that both the TBH and TBM groups harbor a limited number of low-abundance characteristic microbial communities, resulting in differences in KEGG pathways enriched at TB dose levels. As detailed in the experimental results, the shared signals between the two groups include the significant activation of caffeine metabolism and a trend toward enrichment of immune-related pathways, driven by Dg-series lipids and histamine. The inter-group differences are as follows: the TBH group exhibits caffeine metabolism as the sole significantly enriched pathway, accompanied by a broad trend toward enrichment of neuro-endocrine pathways. In contrast, the TBM group demonstrates significant enrichment of both caffeine metabolism and histidine metabolism, along with overall inhibition of tryptophan metabolism. It should be noted that this study focused on the enrichment of intestinal metabolic pathways without targeted validation of key metabolites or pathway activities, limiting our ability to define the precise molecular mechanisms. Future studies should further investigate this by integrating fecal microbiota transplantation, targeted metabolomics, and transcriptomics.

## 5. Conclusions

In summary, this study demonstrates that TB alleviated CTX-induced immunosuppression in mice, restoring both systemic and intestinal immune parameters. Beneficial gut bacteria, including *Lactobacillus* and *Candidatus Saccharimonas*, were enriched alongside a remodeled microbiota–metabolite network. Caffeine and histidine metabolism, as well as immune signaling pathways linked to Dg-series lipid metabolites, may contribute to these effects, although the associations observed here are correlative and need further validation. Given its natural origin and favorable safety profile, TB could serve as an immunomodulatory ingredient in food and pharmaceutical products, but its efficacy and mechanisms in humans are still unknown.

## Figures and Tables

**Figure 1:**
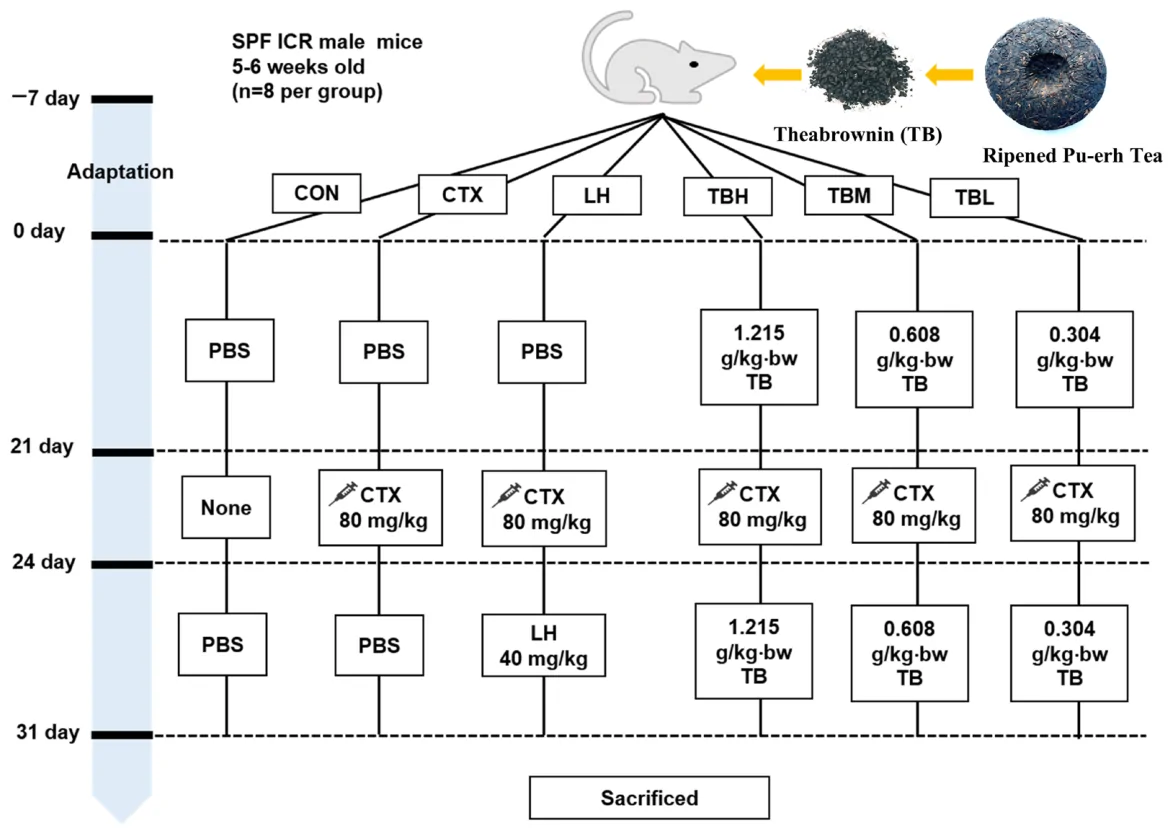
Animal experiment design scheme.

**Figure 2:**
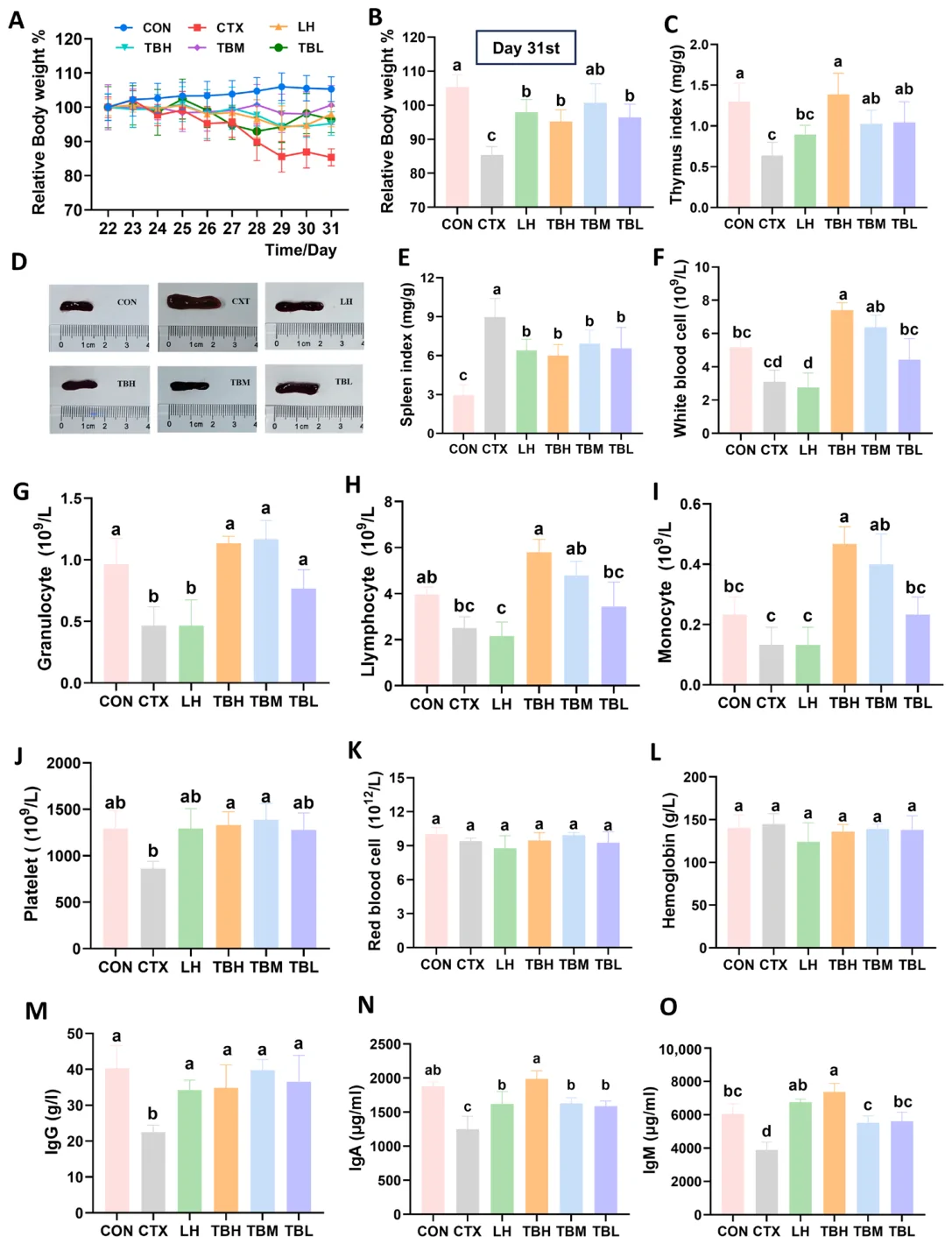
Effects of TB on body weight, organ indices, and blood parameters in CTX-induced mice. (**A**) Relative body weight % (Day 22nd–31st) (n = 8); (**B**) body weight comparison in mice at Day 31st (n = 8); (**C**) thymus indices (n = 8); (**D**) photographs of isolated spleens from each group; (**E**) spleen indices (n = 8); (**F**–**L**) the content of peripheral blood cells (n = 4); (**M**–**O**) the level of immunoglobulins (IgG, IgA, IgM) in serum (n = 4). Lowercase letters denote significant differences between groups (*p* < 0.05).

**Figure 3:**
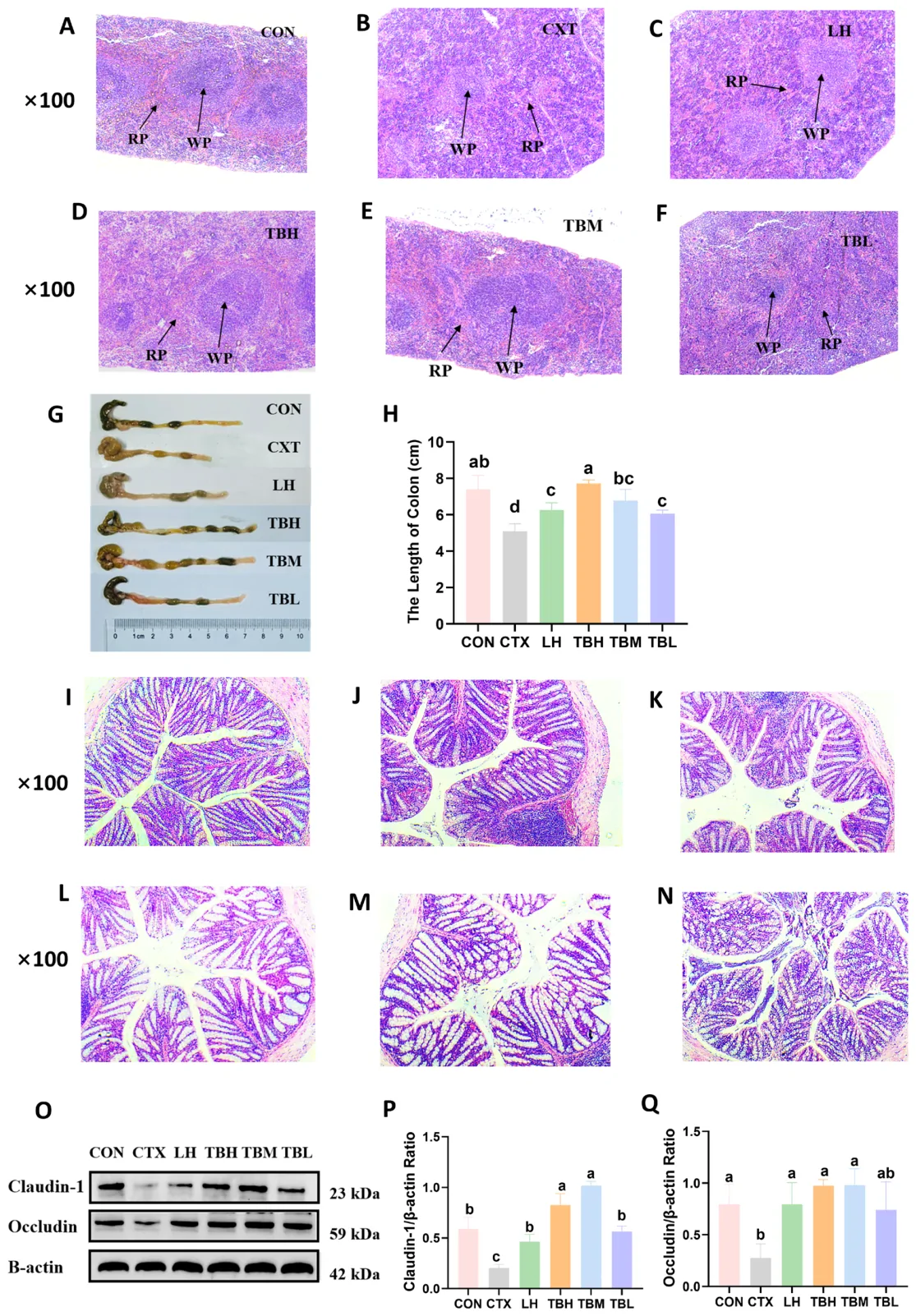
Effects of TB on the spleen and colon tissues. (**A**–**F**) H&E sections of spleen (n = 3); (**G**,**H**) length of colon (n = 8); (**I**–**N**) H&E sections of colon (n = 3); (**O**–**Q**) expression levels of claudin-1 and occludin in the colon (n = 3). Lowercase letters denote significant differences between groups (*p* < 0.05).

**Figure 4:**
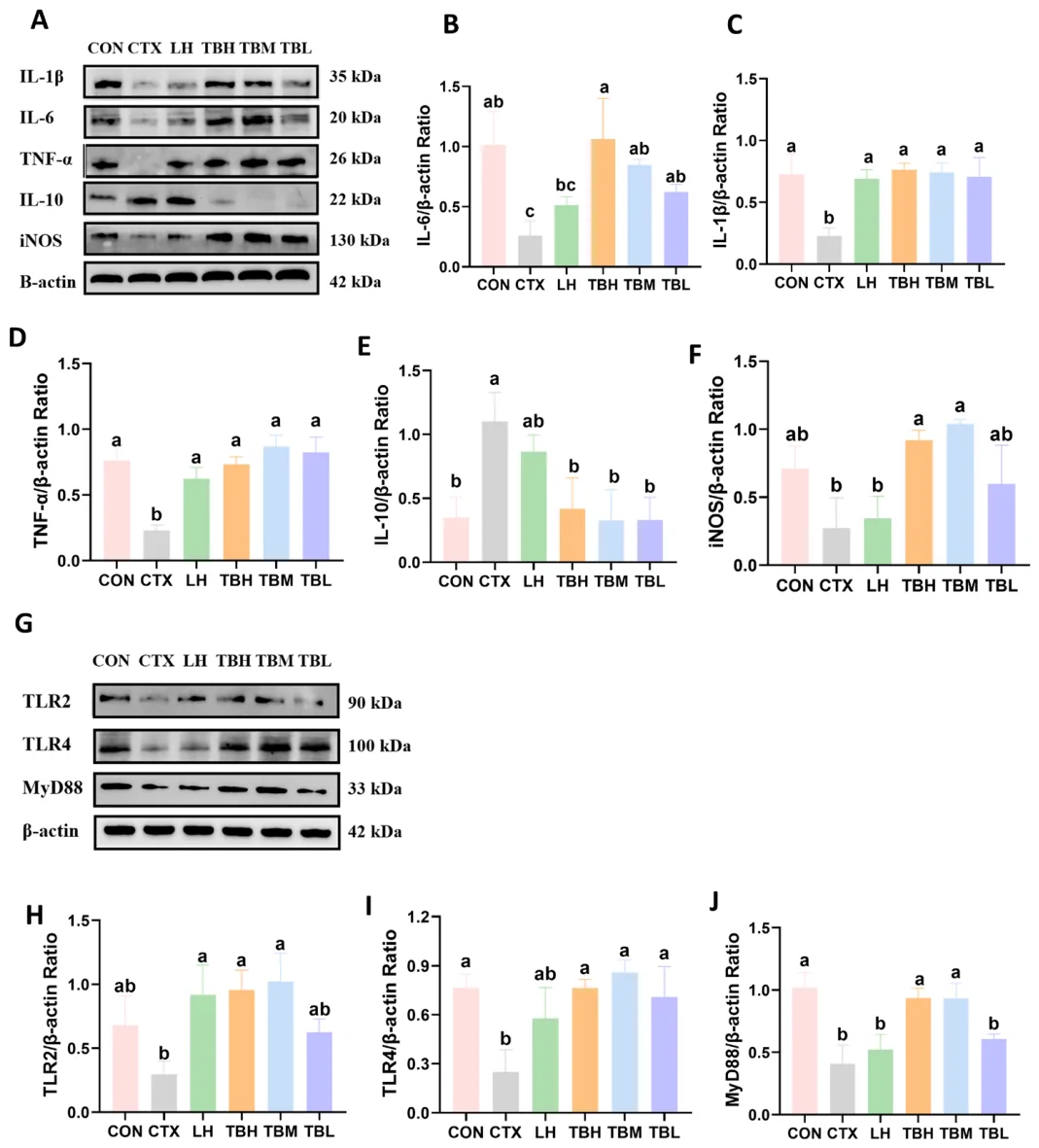
(**A**–**F**) Effects of TB on the distribution of inflammatory mediators in the colon (n = 3); (**G**–**J**) effects of TB on the distribution of TLR2, TLR4, and MyD88 in the colon (n = 3). Lowercase letters denote significant differences between groups (*p* < 0.05).

**Figure 5:**
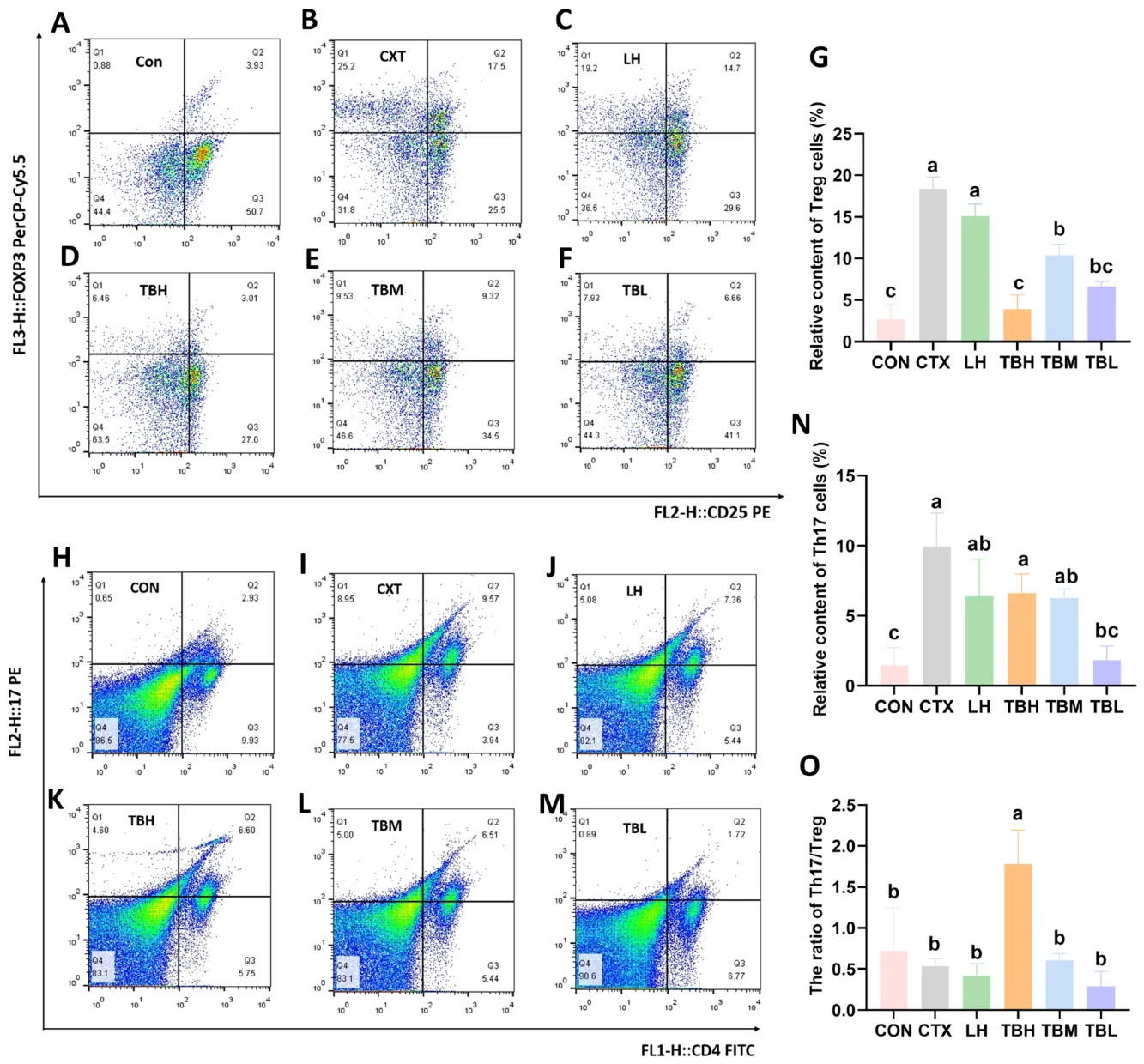
Effects of TB on the differentiation of CD4^+^ T cells in the colon. (**A**–**G**) The differentiation of Tregs (n = 3); (**H**–**N**) the differentiation of Th17 (n = 3); (**O**) the ratio of Th17/Treg (n = 3). Lowercase letters denote significant differences between groups (*p* < 0.05).

**Figure 6:**
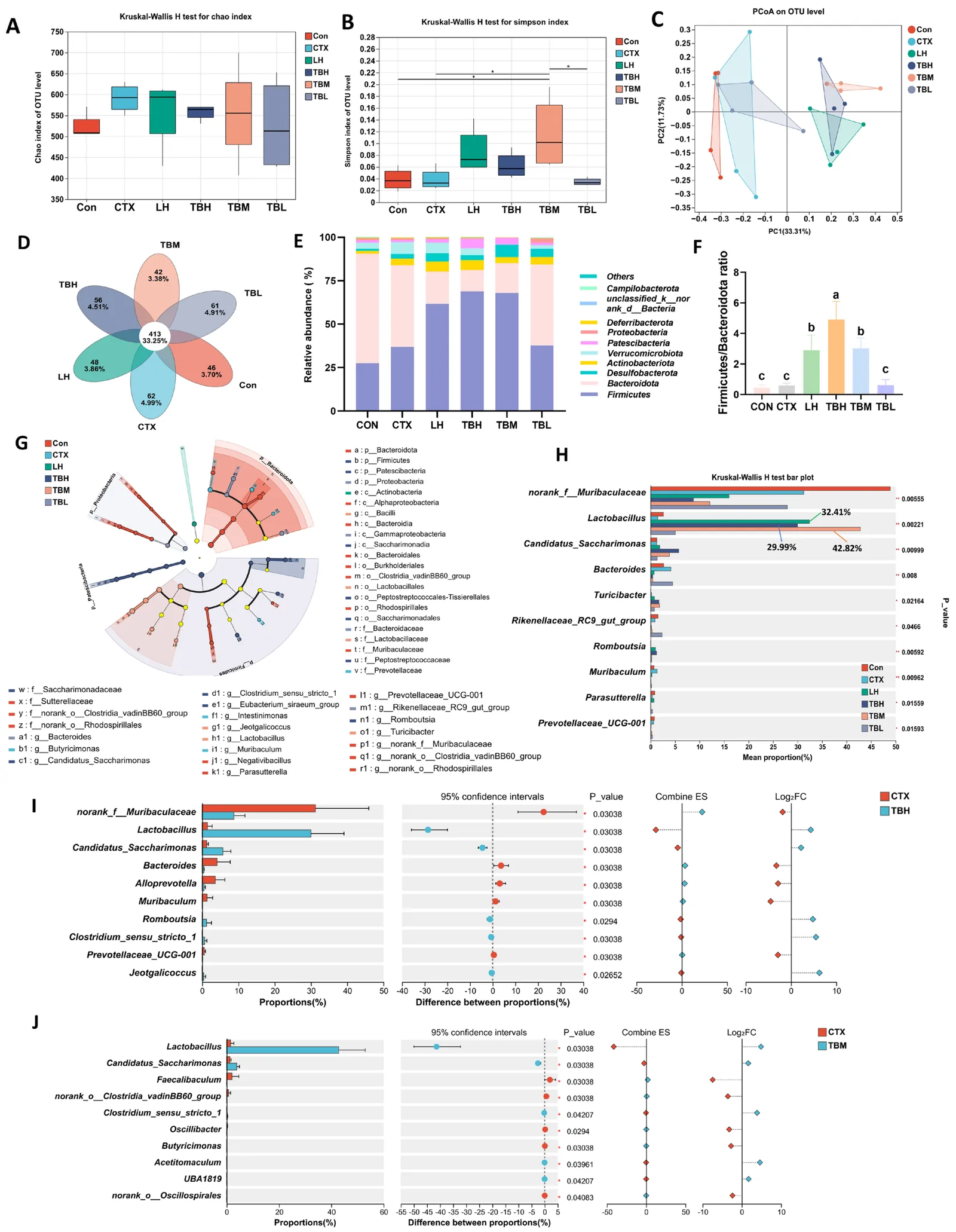
TB regulated the gut microbiota of CTX-induced mice (n = 4). (**A**) Chao 1 index; (**B**) Simpson index; (**C**) PCoA; (**D**) Venn diagram; (**E**) at the phylum level, the top 10 with the highest abundance; (**F**) the ratio of F/B (Lowercase letters denote significant differences between groups (*p* < 0.05)); (**G**) the evolutionary branching relationships among microbial communities across groups; (**H**) at the genus level, the top 10 with the highest abundance; (**I**) significant differences between CTX and TBH groups at genus level. (**J**) Significant differences between CTX and TBM groups at genus level (Wilcoxon rank-sum test for (**I**,**J**)). (Kruskal–Wallis H test, **: *p* < 0.01, *: *p* < 0.05).

**Figure 7:**
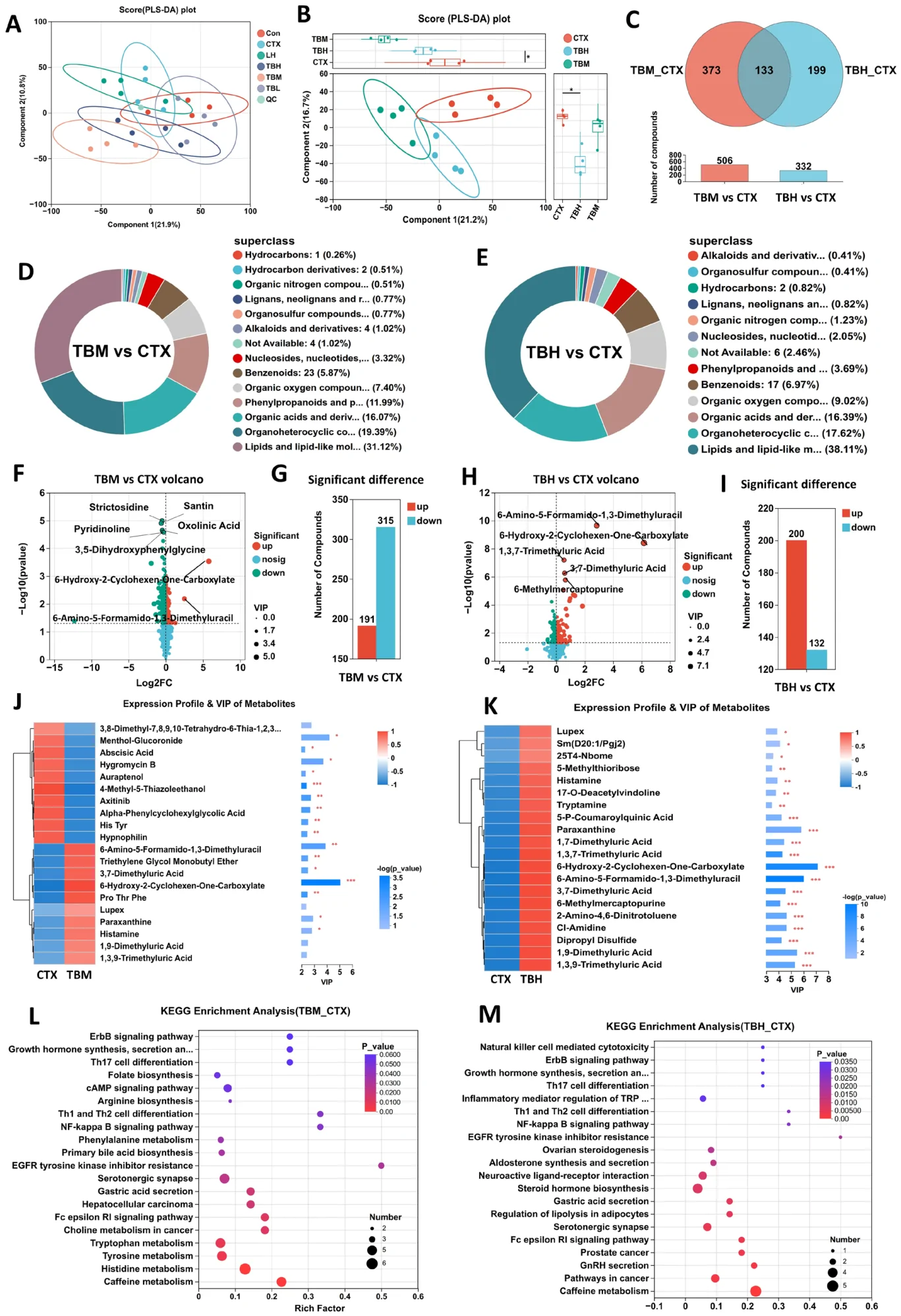
The regulatory effect of TB on CTX-induced intestinal metabolites in mice (n = 4). (**A**) Assessment of metabolic differences in all groups by PLS-DA; (**B**) assessment of metabolic differences in CTX, TBM, and TBH groups by PLS-DA; (**C**) Venn diagram indicates the total number of differential metabolites in CTX compared with TBH and TBM; (**D**) the altered metabolites in TBM vs. CTX; (**E**) the altered metabolites in TBH vs. CTX group; (**F**,**G**) volcano plots of metabolites from the TBM vs. CTX comparison; (**H**,**I**) volcano plots of metabolites from the TBH vs. CTX comparison; (**J**) the VIP scores of differential metabolites in TBM vs. CTX group (VIP > 1 and *p* < 0.05); (**K**) the VIP scores of differential metabolites in TBH vs. CTX group (VIP > 1 and *p* < 0.05); (**L**) the top 20 enriched KEGG pathways of TBM vs. CTX group; (**M**) the top 20 enriched KEGG pathways of TBH vs. CTX group. (*** *p* < 0.001, ** *p* < 0.01, * *p* < 0.05).

**Figure 8:**
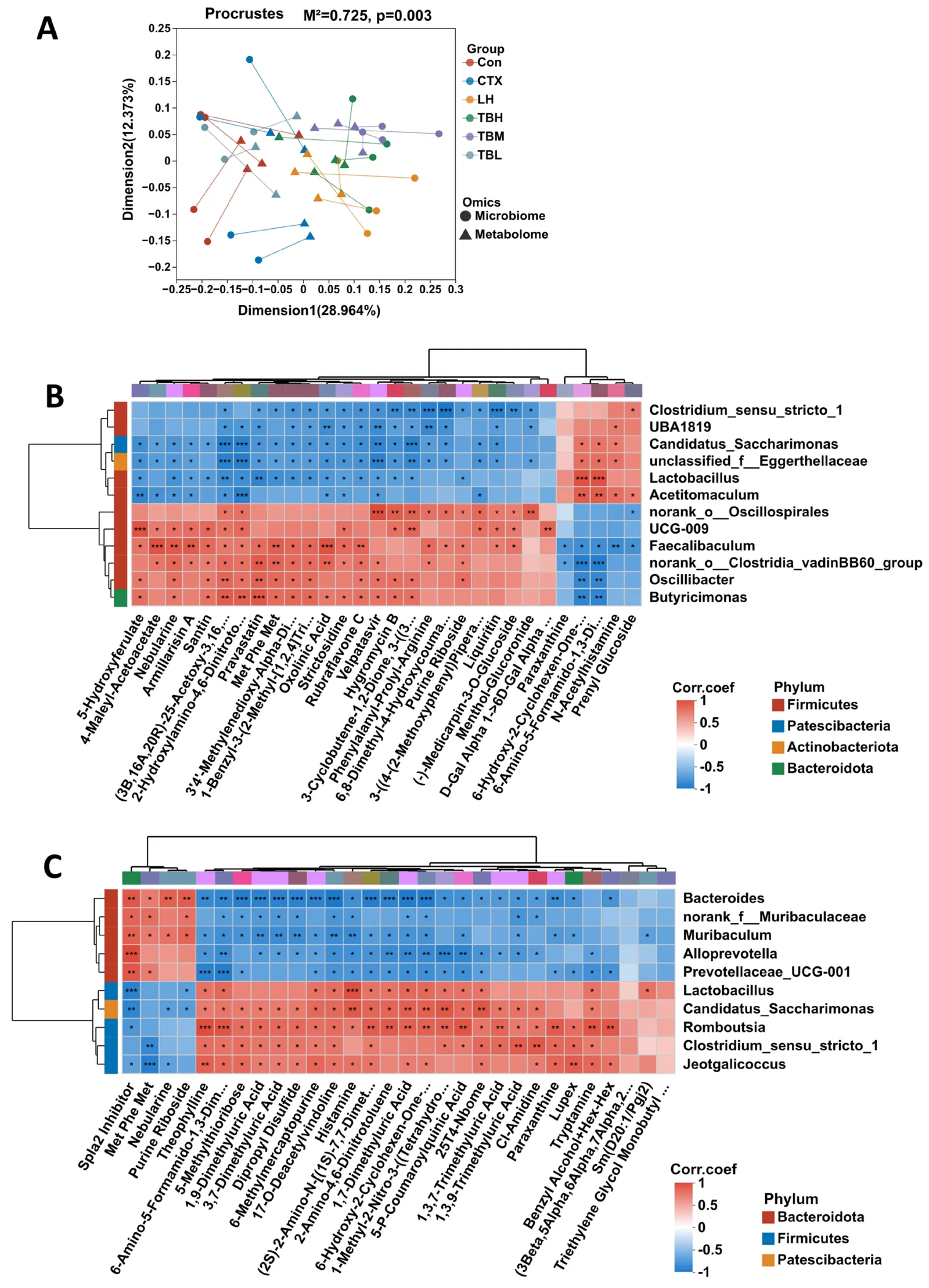
Correlation analysis of metabolomic and microbiome data. (**A**) Gut microbiome and metabolome profiles by Procrustes analysis; (**B**) Spearman correlation heatmap of TBM Vs. CTX group; (**C**) Spearman correlation heatmap of TBH Vs. CTX group. (*** *p* < 0.001, ** *p* < 0.01, * *p* < 0.05).

## Data Availability

The original contributions presented in this study are included in the article. Further inquiries can be directed to the corresponding authors.
